# Leptospirosis Manifesting as HELLP (Hemolysis, Elevated Liver Enzymes, and Low Platelets) Syndrome: A Rare Case of Leptospirosis During Pregnancy

**DOI:** 10.7759/cureus.39083

**Published:** 2023-05-16

**Authors:** Twisha Patel, Sandhya Pajai, Nidhi Patel, Parth Patel

**Affiliations:** 1 Obstetrics and Gynaecology, Jawaharlal Nehru Medical College, Datta Meghe Institute of Higher Education & Research, Wardha, IND; 2 Medical Education and Simulation, Sumandeep Vidyapeeth, Vadodra, IND

**Keywords:** acute fatty liver of pregnancy, spirochete, zoonotic infection, hellp syndrome, leptospirosis

## Abstract

Pregnancy is characterized by a reduced immune response, making pregnant women more susceptible to infections. We present a case of a 24-year-old woman in her second pregnancy who arrived at the hospital at 36 weeks gestation in active labor. The patient had received regular antenatal care including routine prenatal check-ups, screenings, and appropriate vaccinations. She complained of abdominal pain for 5-6 hours, sudden onset of hematuria, and a history of low-grade fever for two days. Physical examination revealed paleness, grade three pedal edema, and elevated blood pressure. Diagnostic tests showed mild anemia, thrombocytopenia, proteinuria, elevated liver enzymes, and kidney dysfunction. The patient was admitted to the labor ward, and a tentative diagnosis of hemolysis, elevated liver enzymes, and low platelets (HELLP) syndrome was made. Shortly after arrival, she spontaneously delivered a healthy baby. However, post-delivery, her fever profile indicated the presence of leptospira IgM antibodies, leading to a diagnosis of leptospirosis mimicking HELLP syndrome. Immediate medical treatment resulted in symptom resolution within two weeks and normal biochemical values within a month. Leptospirosis, caused by the gram-negative spirochete bacteria leptospira, is a zoonotic infection rarely observed during pregnancy and can be misdiagnosed due to its atypical presentation. It can mimic other pregnancy-related conditions such as viral hepatitis, obstetric cholestasis, HELLP syndrome, and acute fatty liver of pregnancy. Early detection and treatment are crucial as this disease can have serious consequences for both the mother and fetus. Therefore, leptospirosis should be considered a potential differential diagnosis, particularly in endemic areas.

## Introduction

Leptospirosis, a zoonotic disease, is caused by spirochete bacteria of the Leptospira genus [1]. It is a global occurrence in endemic regions, primarily in tropical climates. Outbreaks of this disease tend to occur following heavy rainfall or floods in areas with poor sanitation conditions. In India, it is particularly endemic in South Indian states such as Chennai in Tamil Nadu, Gujarat, Maharashtra, Karnataka, and Andaman Nicobar [2]. The primary mode of transmission is through contact with urine from infected animals. Humans can contract the disease by ingesting water or food contaminated with this urine. Human-to-human transmission is extremely rare. The incubation period for leptospirosis is typically two to thirty days, and most cases manifest within five to fourteen days after exposure. In pregnant women, leptospiral infection can lead to severe maternal morbidity and fetal complications including miscarriage, intrauterine death, and stillbirths during the late trimester [3]. The case fatality rate among patients with severe illness ranges from 5% to 15% [4].

Leptospirosis presents with a wide range of symptoms, varying from nonspecific acute febrile illness to a combination of renal and liver failure, as seen in Weil's disease [5]. Common symptoms include headache, fever, myalgia (especially in the calves and lower back), conjunctival hemorrhages, nausea, vomiting, diarrhea, abdominal pain, cough, and occasionally, skin rashes. Severe cases may exhibit jaundice, renal failure, pulmonary hemorrhage, meningitis, and hemodynamic collapse [6].

## Case presentation

**Figure 1:**
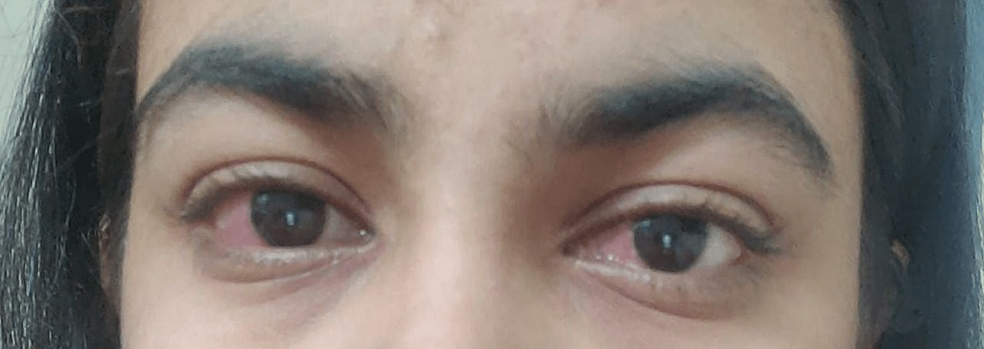
Characteristic conjunctival suffusion as seen in leptospirosis

**Figure 2:**
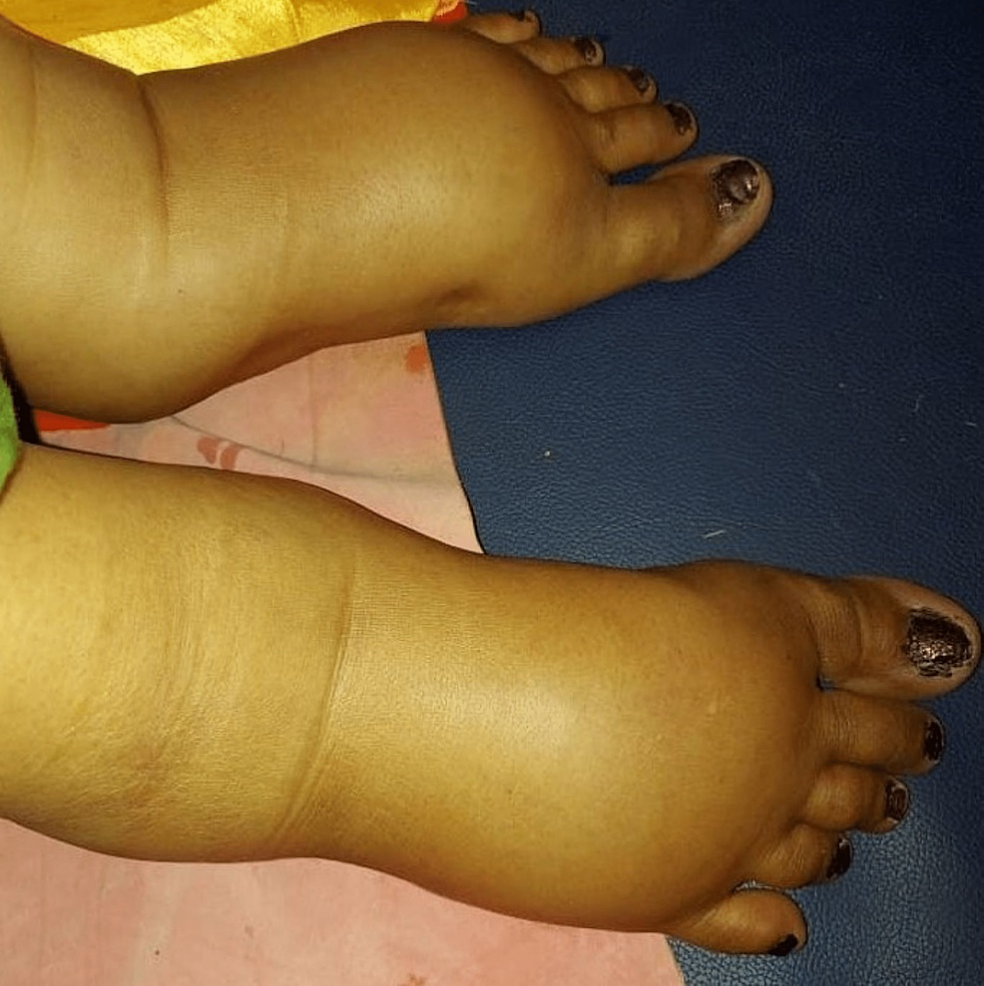
Grade 3 bilateral pedal pitting oedema

A 24-year-old woman who had two pregnancies resulting in one live birth, currently at 36 weeks of gestation, presented to the emergency department of a tertiary care health center in Maharashtra, India. She complained of abdominal pain that was colicky and radiated to her back and thigh. The pain was present for 5-6 hours and was accompanied by a low-grade fever for the past two days. This morning, she also experienced a sudden onset of hematuria, prompting her visit to a private hospital in Yavatmal, where she was subsequently referred to a tertiary care health center for further management. The patient reported redness in her eyes for the past two days and exhibited grade 3 pitting pedal edema (Figures 1-2). She denied experiencing symptoms commonly associated with impending eclampsia, such as headache, blurred vision, or epigastric pain.

Upon examination, her body temperature was 100.2°F, her pulse rate was 108 beats per minute, and her blood pressure was 140/92 mmHg in the left lateral supine position. The patient appeared pale, but there was no evidence of icterus. Generalized myalgia was present. Cardiovascular and respiratory examinations were unremarkable. On abdominal examination, the uterus corresponded to 34 weeks of gestation, and a regular, rhythmic fetal heart rate of over 144 beats per minute was observed. The fetal head was felt to be engaged during the abdominal examination, and strong uterine contractions lasting 35-40 seconds were noted. Vaginal examination revealed the cervix to be dilated 7-8 cm, fully effaced, with the vertex at +1 station and bulging membranes.

The patient was transferred to the labor ward and was put on a continuous cardiotocography monitor. All routine investigations, urine routine microscopy, and fever profile for malaria, dengue, leptospirosis, and widal were sent. The urine protein dipstick was +1, and gross hematuria was present. Vitals were monitored. One stat dose of tab labetalol 100 mg per oral was administered; IV antibiotics were started. Two unit-packed red cells were arranged for transfusion. Within 15 minutes, a bag of membranes ruptured spontaneously at a fully dilated, effaced cervix, and draining liquor was meconium stained. The patient delivered vaginally in the presence of a pediatrician with all resuscitative measures. Liberal episiotomy was given, and a female child of 1.9 kg was born and cried immediately after birth. The baby was taken to NICU given the preterm delivery with low birth weight and metabolic syndrome-like symptoms (MSL). The third stage was actively managed, and 800 mcg of misoprostol was given per rectally to prevent postpartum hemorrhage (PPH). Foley catheterization was done, and 100 ml of concentrated blood-tinged urine was passed. Inj lasix 10 mg IV was administered. Fluids were started at 100ml/hr, and output was monitored. Vitals were monitored, and episiotomy was sutured in layers. The cervix and vagina were explored for tears, and a botroclot pack was inserted. The patient was kept in a high-risk unit. One packed cell volume (PCV) was transfused slowly post-delivery under lasix cover. Pack was removed after six hours, and bleeding per vaginum was monitored. 

Based on her clinical features, our first impression was hemolysis, elevated liver enzymes, and low platelets (HELLP) syndrome. The patient was evaluated for viral causes of fever and was found negative. She had mild direct hyperbilirubinemia with mildly elevated liver enzymes and deranged renal markers, and it was further associated with myalgia, conjunctival suffusion, and new onset of macroscopic hematuria. Her laboratory investigations are summarized in Table 1.

**Table 1:** Laboratory profile of the patient

Name of Investigation	Patient Value
Complete Blood Count	
Haemoglobin	9.2 gm/dl
Total Leucocyte Count	18000/cumm
Peripheral smear	Microcytic, hypochromic with no malarial parasite
International Normalized Ratio (INR)	1.08
Liver Function Test	
Aspartate Aminotransferase	54 U/L
Alanine Transferase	66 U/L
Kidney Function Test	
Serum creatinine	1.2 mg/dl
Fever profile	
Leptospira IgM	Positive

**Figure 3:**
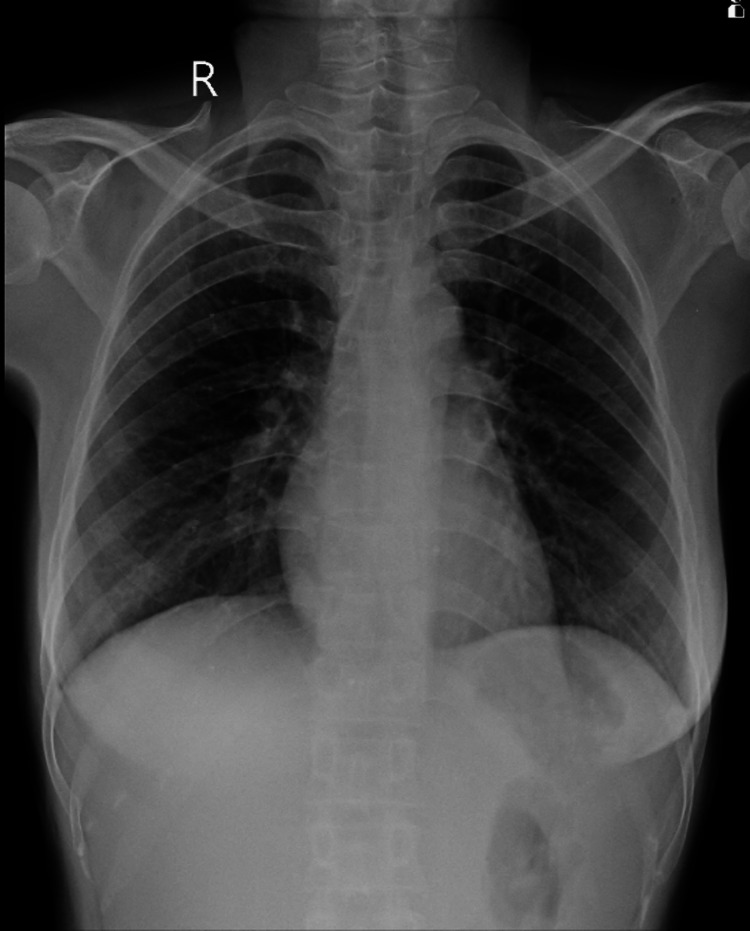
X-ray of the chest showing bilateral peripheral lower lobe opacity with increased vascular markings

An ultrasound was performed to investigate the cause of hematuria, and the results were within normal limits. A chest X-ray revealed mild bilateral lower lobe opacity with increased vascular markings (Figure 3). The leptospira IgM test, conducted using the enzyme-linked immunosorbent assay (ELISA) method, yielded a positive result. The patient consulted with a general physician and a nephrologist, who initiated treatment consisting of intravenous doxycycline, a third-generation cephalosporin, and other supportive measures. On the second day after vaginal delivery, the hematuria resolved. The baby's leptospira test came back negative. The patient was advised to refrain from breastfeeding until her IgM titers disappeared, reducing the risk of transmission to the neonate. Over time, she demonstrated clinical improvement, and her liver and kidney function returned to nearly normal levels by the eighth day. The baby was discharged from the NICU on the seventh day, while the patient herself was discharged on the fifteenth day. Upon discharge, the patient received counseling on preventing leptospira and contraception.

## Discussion

Leptospira infection during pregnancy is rare. However, if an infection does occur during pregnancy, it can be life-threatening, necessitating immediate diagnosis, and treatment. Clinically, leptospirosis manifests in two phases. The initial phase is characterized by the onset of symptoms, while the second phase, known as the immunogenic phase, is marked by the presence of IgM antibodies [4]. If leptospirosis in pregnancy is identified and treated promptly, along with thorough monitoring of the fetus, termination of pregnancy is not indicated. Infections contracted during the first trimester may lead to spontaneous abortions [5], and even a mild infection in the mother near term can result in congenital leptospirosis in the infant, as the bacterium can be transmitted through the placenta [7]. This elevates the risk of stillbirth and intrauterine death [8]. In a case series by Carles et al., neonatal mortality and the risk of abortion were reported in over 50% of pregnant patients infected with leptospirosis [9]. Transmission of the bacterium is also known to occur through breastfeeding, necessitating the suspension of breastfeeding due to an active infection [10].

The most commonly reported infection during pregnancy is typically asymptomatic, while approximately 90% of symptomatic patients experience mild disease and fully recover [5]. Symptoms can vary and include non-specific acute febrile illness, headache, and chills accompanied by fever, abdominal pain, muscle pain, diarrhea, loss of appetite, skin rash, swollen lymph nodes, enlarged liver, and enlarged spleen. Conjunctival suffusion, a distinctive feature of leptospirosis, usually manifests on the third or fourth day of illness. The presence of circulating antibodies can also result in complications such as nephritis, hepatic failure, myocarditis, aseptic meningitis, and pulmonary hemorrhage [11].

The diagnosis of leptospirosis is conducted using serological tests such as the agglutination test and ELISA. Confirmation of the diagnosis is achieved through culture. Laboratory findings are nonspecific and include elevated total leukocyte count (TLC), decreased platelet count, direct hyperbilirubinemia, as well as abnormal hepatic and renal function tests. In advanced stages, coagulation dysfunction may also manifest [8].

The management of leptospirosis is based on the symptomatology exhibited by patients. Mild cases can be effectively treated on an outpatient basis with comprehensive fetal assessment. On the other hand, severe cases and infections occurring near term require IV antibiotics and close monitoring of fetal well-being. Table 2-3 provides a summary of the management approach for leptospirosis [6,12].

**Table 2:** Management of mild disease FDA: Food and Drug Administration, BD: Twice Daily, OD: Once Daily, PO: Per Oral

Drug	Dosage	Category of drug (according to FDA)	Pregnancy use
Doxycycline	100 mg orally BD PO x 7 days	D	Not recommended
Azithromycin	500 mg orally OD POx 3 days	B	Can be used
Amoxicillin	25-50 mg/kg PO x 7 days	B	Can be used

**Table 3:** Management of severe disease FDA: Food and Drug Administration, IV: Intravenous

Drug	Dosage	Category of Drug (according to FDA)	Pregnancy use
Penicillin	1.5 MIU IV x 6 hourly x 7 days	B	Can be used
Doxycycline	100 mg IV 12 hourly x 7 days	D	Not recommended but can be used in severly ill patients or in endemic areas when benefit > risks
Ceftriaxone	1-2 g IV Once daily x 7 days	B	Can be used
Cefotaxime	1gm IV x 6 hourly x 7 days	B	Can be used

## Conclusions

In conclusion, leptospira infection poses a diagnostic challenge in pregnant individuals due to its varied clinical presentation and underdiagnosis in endemic areas. The impact on maternal and fetal health is significant. The disease can often mimic other conditions, making accurate diagnosis crucial, such as viral hepatitis, obstetric cholestasis, HELLP syndrome, and acute fatty liver during pregnancy. Although rare, the fatality rates can range from 0%-15%, with higher rates observed in icteric leptospirosis. However, early detection and proper management greatly improve the chances of a favorable outcome. It is imperative to conduct further studies to enhance our understanding of this illness. Additionally, it is crucial to reduce its incidence by emphasizing preventive measures such as environmental sanitation, promoting good hygiene practices, and raising awareness about leptospira infection in susceptible regions. By adopting these measures, we can strive to mitigate the burden of this disease and protect the health of pregnant individuals and their unborn babies.
